# In Vitro Activity of Monofunctional Pt-II Complex Based on 8-Aminoquinoline against Human Glioblastoma

**DOI:** 10.3390/pharmaceutics13122101

**Published:** 2021-12-07

**Authors:** Valentina Coccè, Isabella Rimoldi, Giorgio Facchetti, Emilio Ciusani, Giulio Alessandri, Lucia Signorini, Francesca Sisto, Aldo Giannì, Francesca Paino, Augusto Pessina

**Affiliations:** 1 Department of Biomedical, Surgical and Dental Sciences, University of Milan, CRC StaMeTec, 20133 Milan, Italy; valentina.cocce@unimi.it (V.C.); cisiamo2@yahoo.com (G.A.); francesca.sisto@unimi.it (F.S.); aldo.gianni@unimi.it (A.G.); francesca.paino@unimi.it (F.P.); 2 Department of Pharmaceutical Science, University of Milan, Via Golgi 19, 20133 Milan, Italy; isabella.rimoldi@unimi.it; 3 Laboratory of Clinical Pathology and Neurogenetic Medicine, Fondazione IRCCS Neurological Institute Carlo Besta, 20133 Milan, Italy; emilio.ciusani@istituto-besta.it; 4 Department of Biomedical, Surgical and Dental Sciences, University of Milan, 20133 Milan, Italy; lucia.signorini@unimi.it; 5 Maxillo-Facial and Dental Unit, Fondazione Ca’ Granda IRCCS Ospedale Maggiore Policlinico, 20122 Milan, Italy

**Keywords:** mesenchymal stromal cells, glioblastoma, monofunctional Pt-II complex, cisplatin

## Abstract

A new cationic Pt(II) complex bearing 8-aminoquinoline as chelating ligand (called Pt-8AQ) was evaluated against two human carcinomas, one mesothelioma, and three glioblastoma cell lines. The in vitro comparison to the clinically approved CisPt showed a minor activity of Pt-8AQ against carcinoma and mesothelioma, whereas a significant activity of Pt-8AQ was observed on the proliferation of the three glioblastoma cell lines (U87-MG IC_50_ = 3.68 ± 0.69 µM; U373-MG IC_50_ = 11.53 ± 0.16 µM; U138-MG IC_50_ = 8.05 ± 0.23 µM) that was higher than that observed with the clinically approved CisPt (U87-MG IC_50_ = 7.27 + 1.80 µM; U373-MG IC_50_ = 22.69 ± 0.05 µM; U138-MG IC_50_ = 32.1 ± 4.44 µM). Cell cycle analysis proved that Pt-8AQ significantly affected the cell cycle pattern by increasing the apoptotic cells represented by the sub G0/G1 region related with a downregulation of p53 and Bcl-2. Moreover, an NMR investigation of Pt-8AQ interaction with 9-EtG, GSH, and Mets7 excluded DNA as the main target, suggesting a novel mechanism of action. Our study demonstrated the high stability of Pt-8AQ after incubation at 37 °C and a significant antineoplastic activity on glioblastomas. These features also make Pt-8AQ a good candidate for developing a new selective advanced cell chemotherapy approach in combination with MSCs.

## 1. Introduction

Cancer is undoubtedly one of the most diffuse and challenging arrays of diseases of the modern era, accounting for millions of deaths per year. Many important achievements in this field have been realized thanks to the progression in the development of new therapeutics, in which platinum(II) complexes play a prominent role. Despite cisplatin finding application in a wide range of clinical treatments, especially on solid tumors, its intrinsic or acquired side effects still represent a significant drawback that researchers have been trying to bypass with the synthesis of alternative platinum compounds [1,2].

All the three currently approved platinum (II) drugs, i.e., cisplatin, carboplatin, and oxaliplatin, have DNA as the main target; in fact, the platination of cellular DNA occurred in a major amount compared to genomic one, once inside the cell. In addition, all the platinum(II) complexes possess a high affinity for sulfur-containing molecules (glutathione, cysteines, and methionines’ containing enzymes, etc.), and their interaction represents both a resistance mechanism, carried out by the cells against platinum drugs, and a delivery system for the complex for rightly interacting with cellular DNA [3].

**Scheme 1:**
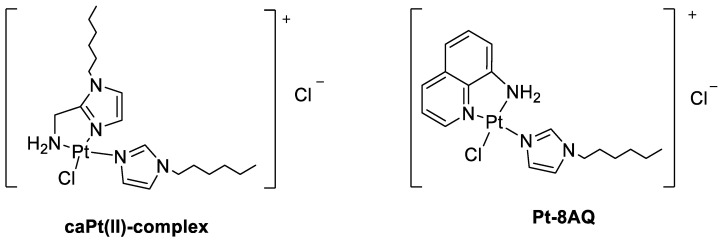
Cationic platinum(II) complexes: caPt(II) complex and Pt-8AQ.

The type of adducts and the amount of DNA platination strictly depends on the structure of the compounds in which the type of ligand plays an important role in discriminating the main target [4,5,6]. As reported in our previous works, the structural and electronic modifications of the chelating ligand, a methylamino-imidazole (caPt(II)-complex) [7], or an 8-aminoquinoline (Pt-8AQ), [8] coordinated to the platinum metal center, allowed for the obtaining of possible different behavior of the complexes in targeting and interacting with the biological counterpart inside the cell [9] (Scheme 1).

In this work, we focused our attention on the characteristics of 8-aminoquinoline as ligand, known for being involved in many pharmacological pathways [10,11], as well as its capability to influence the biological behavior of the corresponding platinum complex.

In particular, the present preliminary study evaluated the stability and the activity of the new Pt-8AQ against two human carcinoma cell lines, one mesothelioma, and three glioblastomas. Further studies have been addressed to the human glioblastomas in which this quinoline derivative revealed a significant antineoplastic activity with a mechanism of cytotoxicity different from that known for cisplatin used as control in our study.

## 2. Materials and Methods

### 2.1. Drugs and Tumor Cells

Rates of cisplatin (CisPt) and caPt(II) complex (complex 2c; called Pt-8AQ) [7] were prepared at the concentration of 4 mg/mL in dimethyl sulfoxide (DMSO; Sigma-Aldrich, St. Louis, Missouri, USA). Working solutions were freshly prepared according to the experimental design by serial dilutions in complete culture medium. The *in vitro* activity of CisPt and Pt-8AQ was tested against six tumor cell lines: glioblastoma cell lines U87-MG [12] (expressing wild-type p53), U373-MG [13] and U138-MG [14] (expressing endogenous mutant p53), pancreatic adenocarcinoma cell line CFPAC-1 [15], adenocarcinoma MCF-7 [16], and biphasic mesothelioma MSTO-211H. [17] Cells lines were maintained by 1:5 weekly dilution in Roswell Park Memorial Institute (RPMI) medium (MSTO-211H), Dulbecco’s modified Eagle’s medium (DMEM LG) (U87-MG, U373-MG, U138-MG, and MCF-7), and Iscove’s modified Dulbecco’s medium (IMDM) (CFPAC-1), supplemented by fetal bovine serum (FBS) 10%. All reagents for culture were provided by Euroclone, Pero (MI), Italy.

### 2.2. Proliferation Assay, Cytotoxic Assay, and In Vitro Stability of Drugs

The effect of CisPt and Pt-8AQ against cell proliferation has been studied in 96 multiwell plates (Sarstedt, Nümbrecht, Germany). Briefly, 1:2 serial dilutions of pure drug (from 0.52 to 64 µM CisPt and from 0.27 ± 35.52 µM Pt-8AQ) were prepared in 100 µL of culture medium per well, and then to each well, we added 2000 tumor cells. After 7 days of culture (anti-proliferative assay) at 37 °C and 5% CO_2_, cell growth was evaluated by MTT assay (3-(4,5-dimethyl-2-thiazolyl)-2,5-diphenyl-2-H-tetrazolium), as previously described. [18,19]

The in vitro stability of CisPt and Pt-8AQ was analyzed, maintaining the drugs at 37 °C for 24 h; then, the drugs were tested on U87-MG cells in anti-proliferation assay and compared to fresh drugs. Cytotoxicity assay (24 h at 37 °C, 5% CO_2_) was performed at increasing concentrations of 2.5, 5, 10, and 20 μM. The inhibitory concentration (IC_50_) was determined by applying the Reed and Muench formula [20] using Excel (Microsoft, Inc, Albuquerque, NM, USA).

### 2.3. Interaction of Pt-8AQ with GSH, 9-EtG, and Mets7

^1^H-NMR spectra were recorded on a Bruker DRX Advance 300 MHz equipped with a non-reverse probe. Chemical shifts were expressed as ppm (δ) using the central peak of deuterated solvent as the internal reference. MS analyses were performed by using a Thermo Finnigan (Palmer, Massachusetts, MA, USA) LCQ Advantage system MS spectrometer with an electrospray ionization source and an ‘Ion Trap’ mass analyzer. The MS spectra were obtained by direct infusion of a sample solution under ionization, ESI-positive. Mets7 was prepared using a Liberty Microwave Peptide Synthesizer (CEM Corporation, North Carolina, NC, USA) and purified by preparative RP-HPLC using Jasco BS-997-01 equipment and a DENALI C-18 column from GRACE VYDAC (10 µm, 250 × 22 mm). Two mobile phases were used: A = 95% Water, 5% ACN, 0.1% TFA; B = 95% ACN, 5% Water, 0.1% TFA. The CD spectra were recorded at room temperature using a Jasco J-810 spectropolarimeter and a 0.01 cm quartz cell (Hellma Suprasil, Hellma UK LTD, Essex SS2 6HZ Southend on Sea, United Kingdom).

The binding studies with 9-ethylguanine and GSH were directly conducted in NMR tube mixing 1 eq. of Pt-8AQ in 20% of 0.9 % *w*/*v* NaCl-D_2_O solution in DMSO-d_6_. A total of 1 eq. of 9-ethylguanine (9-EtG) or glutathione (GSH) was added to the solution. The samples were evaluated within 48 h at 4 h intervals by ^1^H-NMR analysis [21].

The binding study with Mets7, synthetized as previously reported [22], was performed by using microwave-assisted peptide synthesis using Fmoc Chemistry. The study was conducted dissolving Mets7 peptide and Pt-8AQ in 50 mM ammonium acetate buffer (pH = 6) to a final concentration of 15 mM. After 5 days, the solution was analyzed by electrospray ionization mass spectrometry (ESI-MS) with the source at 350 °C.

### 2.4. RT-PCR Analysis and Primers

RNA extraction and RT–qPCR: Total RNA was extracted from pellet of U87-MG, U373-MG, and U138-MG (5 × 10^5^ cells) treated with CisPt (10 µM) or Pt-8AQ (10 µM) for 24 h following instructions of PureLink™ RNA Mini Kit (Thermofisher scientific, Waltham, Massachusetts, USA). RNA was treated with DNase to exclude DNA contamination, and then, 1 μg of RNA was reverse-transcribed into cDNA using a QuantiTect Reverse Transcription Kit (Qiagen, Hilden, Germany). Samples were analyzed using real-time quantitative PCR (RT-qPCR). PCR reactions were performed using Rotor-Gene Q cycler (Qiagen), and the amplifications were carried out using the QuantiNova™ SYBR^®^ Green PCR (Qiagen) in a total volume of 20 μL. Real-time PCR was performed using the primer sequences, as shown in Table 1.

Data were analyzed by using the 2^−ΔΔCt^ method to obtain the relative expression level, and each sample was normalized by using GAPDH RNA expression. The experiments were carried out in duplicate, and each data reports the mean value.

### 2.5. Cell Cycle Analysis

For studying cell cycle analysis, U87-MG, U373-MG, and U138-MG cells were seeded at concentration of 20,000 cells/cm^2^ in multiwell 6 plates and treated with CisPt and Pt-8AQ at 10 µM. After 24, 48, and 96 h (37 °C, 5% CO_2_), DNA content for cell cycle phase detection was performed by comparing untreated cells and 24 h treated cells. Cells were suspended in phosphate-buffered saline (PBS) and fixed with 70% (*v*/*v*) ethanol for 1 h at 4 °C. After a further PBS wash, cells were suspended in propidium iodide 50 µg/mL in PBS. Cells were incubated overnight at 4 °C and analyzed by flow cytometry (Navios EX, Beckman Coulter, Brea, CA, USA). At least 10^4^ events per sample were acquired and analyzed using a specific software (Navios EX Softwar, Beckman Coulter). Data are expressed as the mean fluorescence intensity (MFI) of the specific antibody.

### 2.6. Apoptosis/Necrosis in Glioblastoma Cell Lines

The evaluation of apoptosis and necrosis of U87-MG, U373-MG, and U138-MG cells treated with CisPt or Pt-8AQ at 10 µM for 24 h was performed by fluorescence microscope using the Apoptosis/Necrosis Assay Kit (blue, green, red) (Abcam, ab176749, Cambridge, UK), following the manufacturer’s instructions.

The caspase-3 activity in U87-MG, U373-MG, and U138-MG cells was measured by the Caspase-3 Assay Kit (Abcam, ab3940). Briefly, the glioblastoma cells were seeded at a concentration of 2 × 10^4^ cells/cm^2^ (T75), and after 24 h of culture, the cells were treated with Pt-8AQ or CisPt (10 µM) added to medium and incubated for 4 h. At the end of incubation, both adherent and detached cells were harvested and lysed on ice. After centrifugation, the presence of caspase-3 activity in the lysate was examined according to the supplier’s protocols.

### 2.7. Statistical Analysis

Data are expressed as average ± standard deviation (SD). Differences between mean values were evaluated according to Student’s *t*-test performed by GRAPHPADINSTAT program (GraphPad Software Inc., San Diego, CA, USA). *p*-values ≤ 0.05 were considered statistically significant. The linearity of response and the correlation were studied using regression analysis by Excel 2013 software (Microsoft, Inc., Albuquerque, New Mexico, USA).

## 3. Results

### 3.1. In Vitro Antitumor Activity of CisPt and Pt-8AQ on Different Cancer Cell Lines

**Figure 1:**
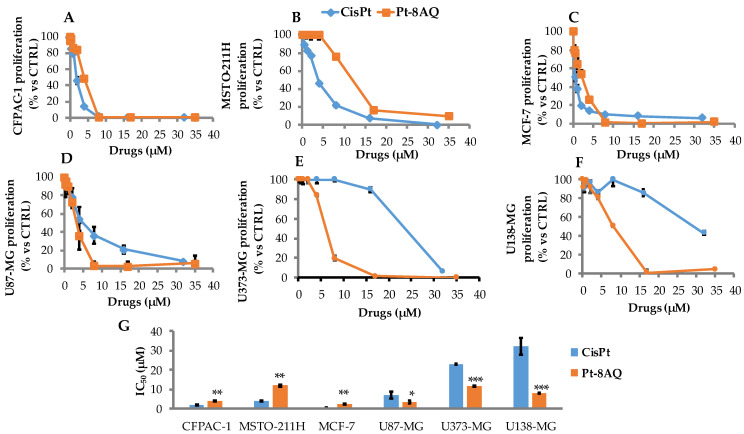
In vitro antitumor activity of CisPt and Pt-8AQ on different cancer cell lines. The effect of increasing concentrations of CisPt or Pt-8AQ was evaluated by a 7 day antiproliferation MTT assay on different tumor cell lines: pancreatic adenocarcinoma CFPAC-1 (**A**), mesothelioma MSTO-211H (**B**), adenocarcinoma MCF-7 (**C**), and three glioblastoma cell lines: U87-MG (**D**), U373-MG (**E**), and U138-MG (**F**). The effect was expressed as a percentage of the optical density measured in cultures that did not receive drugs (100% proliferation). Histogram (**G**) reports the IC_50_ values expressed as mean ± standard deviation (SD) of three independent experiments (* *p* < 0.05; ** *p* < 0.02; *** *p* < 0.001).

The antiproliferative activity of Pt-8AQ molecule was evaluated *in vitro* against six human cancer cell lines in comparison to that of CisPt. As reported in Figure 1, both the drugs produced a dose–response inhibition in all the cancer cell lines. Moreover, Pt-8AQ molecule also showed an important activity on all the cancer cell lines. The CisPt activity was significantly higher for CFPAC-1, MCF-7, and MSTO-211H but not for glioblastoma cell lines U87-MG, U373-MG, and U138-MG. On these tumors, the results showed a higher cytotoxicity of Pt-8AQ (U87-MG IC_50_ = 3.68 ± 0.69 µM; U373-MG IC_50_ = 11.53 ± 0.16 µM; U138-MG IC_50_ = 8.05 ± 0.23 µM) that was statistically significant (*p* < 0.01) with respect to that exerted by CisPt (U87-MG IC_50_ = 7.27+ 1.80 µM; U373-MG IC_50_ = 22.69 ± 0.05 µM; U138-MG IC_50_ = 32.1 ± 4.44 µM).

### 3.2. In Vitro Stability of CisPt and Pt-8AQ to Treatment at 37 °C

**Figure 2:**
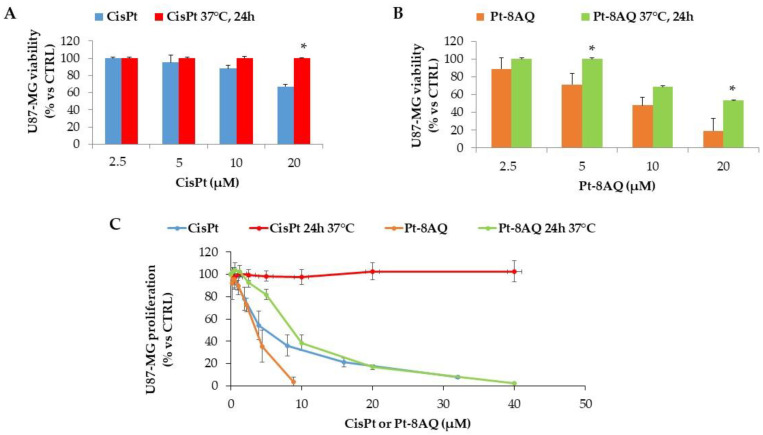
Stability of CisPt and Pt-8AQ on U87-MG. The cytotoxic activity was evaluated after 24 h of treatment with different concentrations of fresh drugs or drugs incubated for 24 h at 37 °C ((**A**) CisPt, (**B**) Pt-8AQ from 2.5 to 20 μM) and expressed as cell viability (% of control cells). (**C**) The effect of increasing concentrations of CisPt or Pt-8AQ was evaluated by a 7 day antiproliferation MTT assay. Fresh prepared drugs or drugs incubated for 24 h at 37 °C were tested. Figures reports mean ± standard deviation (SD) of three independent experiments (* *p* < 0.05).

By using the tumor model of U87-MG, we compared the stability of Pt-8AQ and CisPt to each other by evaluating the anticancer activity of the drugs after their incubation at 37 °C for 24 h. Indeed, this important feature of the two platinum compounds was assessed by determining the dose response inhibiting kinetics (Figure 2). The study showed that after the incubation, both platinum drugs lost their anticancer activity at different extents. We investigated the ability to affect cell viability and cell proliferation by using a standardized MTT assay. In the viability assay, Pt-8AQ displayed an increase of IC_50_ from 9.75 ± 2.10 µM to 20 µM corresponding to a 2.05-fold decreased activity indicating that a significant pharmacological activity was still present (Figure 2B), while the yet low activity of CisPt was completely lost after 37 °C treatment (Figure 2A).

In the antiproliferation assay, Pt-8AQ displayed an increase of IC_50_ from 3.68 ± 0.69 µM (fresh drug) to a value of 8.39 ± 0.79 µM, corresponding to a 2.27-fold decreased activity but indicating that a significant pharmacological activity was still present; after 24 h treatment, no IC_50_ value of CisPt was detectable until the concentration of 70 µM tested (Figure 2C). Even after 6 days of treatment at 37 °C, Pt-8AQ maintained some pharmacological activity with a IC_50_ value of 44.83 ± 1.04 µM (data not reported).

### 3.3. Study of Interaction between Pt-8AQ, GSH, 9-EtG, and Mets7

**Figure 3:**
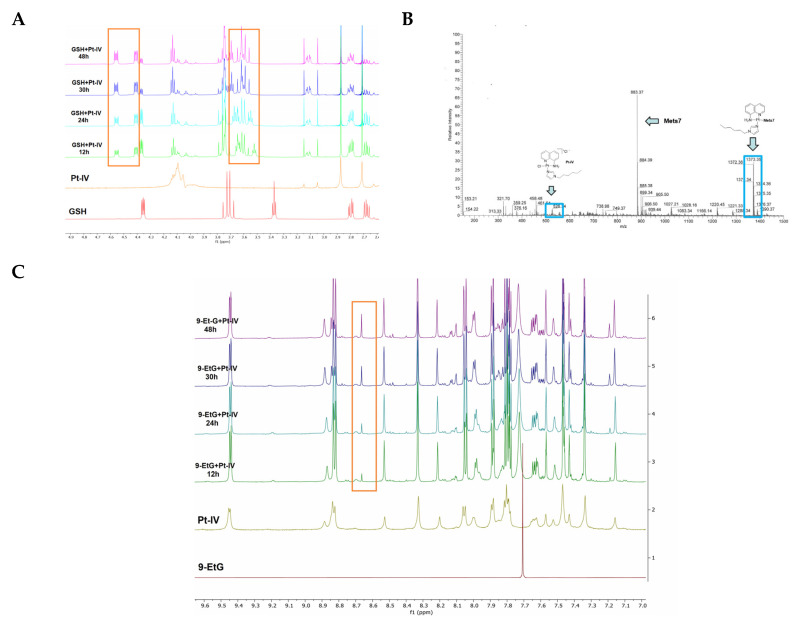
Binding study. (**A**) Binding study between Pt-8AQ and GSH within 48 h. (**B**) ESI-MS spectra of peaks due to the lack of interaction between Mets7 and complex Pt-8AQ after 5 days 1:1 mixture. (**C**) Binding study between Pt-8AQ and 9-EtG within 48 h.

Once inside the cell, one of the most important responsible factors of drug resistance toward platinum complexes is glutathione (GSH), a thiol-containing tripeptide. The binding study between GSH and Pt-8AQ was conducted using 0.9% *w*/*v* NaCl/D_2_O solution in DMSO-d_6_. As shown in the ^1^H-NMR spectrum depicted in Figure 3A, there were two multiplets at 3.51–3.53 and 3.55–3.62 ppm, assigned to the shift of the methylene protons next to the SH group originally present as a multiplet located at 3.35–3.40 ppm. Moreover, two new multiplets at 4.42–4.45 and 4.57–4.60 ppm arose in the spectrum, originally represented as a multiplet at 4.34–4.39 ppm and relative to two methane functions, underlining the interaction between Pt-8AQ and GSH (Figure 3A).

Another recognized source of either tumor cell chemosensitivity or chemoresistance derives from the influence exerted by the membrane transporters and channels, collectively known as the transportome. Among them, copper transporters (CTRs) were involved in cellular uptake and potential platinum sensitivity/resistance as previously reported by our research group. The determination of the physical interaction of Pt-8AQ with the Met7, an octapeptide mimicking the CTR1 transporter, was evaluated, after 5 days, by ESI-MS analysis. A significant number of mono-charged species (Mets7 + Pt-8AQ), corresponding to a monocoordinated complex in a 1:1 stoichiometry, was evinced, in contrast with previously reports for other positive charged platinum complexes (Figure 3B).

In a similar way, the interaction of complex Pt-8AQ with 9-ethylguanine (9-EtG) was studied by ^1^H-NMR spectroscopy to investigate if DNA could be the main target and the probable binding mode between platinum complex and DNA. 9-EtG is generally used as a DNA model base for the physical chemical studies of platinum complexes. The obtained ^1^H-NMR spectrum was characterized by the appearance of a singlet at 8.64 ppm, originally at 7.71 ppm, ascribable to the shift of the H(8) proton in the free 9-EtG upon interaction of the base with Pt-8AQ (Figure 3C).

### 3.4. Cytotoxicity Activity of Pt-8AQ on Glioblastoma Cell Lines

**Figure 4:**
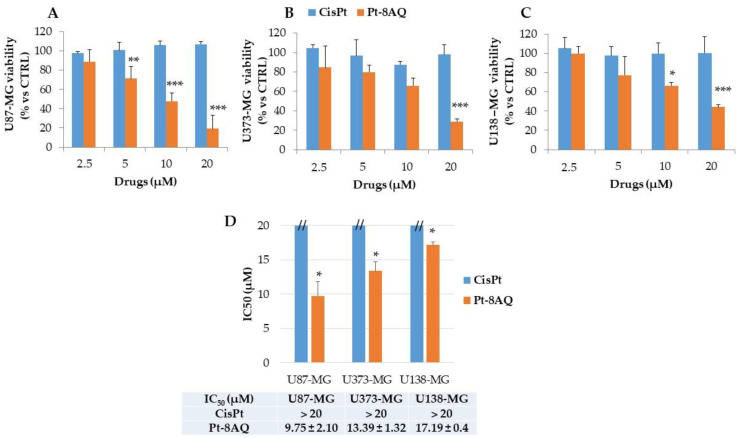
In vitro cytotoxicity of CisPt and Pt-8AQ on glioblastoma cell lines. The cytotoxic activity was evaluated on U87-MG (**A**), U373-MG (**B**), and U138-MG (**C**) after 24 h of treatment with different concentrations of drugs (from 2.5 to 20 μM) and expressed as cell viability (% of control cells). The histogram and box (**D**) reports the IC_50_ values expressed as mean ± standard deviation (SD) of three independent experiments (* *p* < 0.05; ** *p* < 0.02; *** *p* < 0.001).

Besides the antiproliferative effect of the two drugs on U87-MG, U373-MG, and U138 MG cells, we investigated their ability to affect cell viability after 24 h of treatment by using a standardized MTT assay. As reported in Figure 4, by increasing the drug concentration from 2.5 µM to 20 µM, we found that Pt-8AQ was able to impair cancer cells viability with a dose–response kinetics in a percentage of cytotoxicity of 70–80% on U87-MG (Figure 4A) and U373-MG (Figure 4B), and of 60% on U138-MG (Figure 4C) at the dosage of 20 µM. Conversely, CisPt did not significantly affect the viability of cells up to a concentration of 20 µM. In terms of IC_50_ values (Figure 4D), Pt-8AQ was proven to still be active at 24 h on all the glioblastomas, although to different degrees, showing appreciable IC_50_ values ranging from 9.75 µM to 17.19 µM, as reported below in Figure 4D. In the case of CisPt, the IC_50_ values were not detectable, exerting a 10–15% maximal cytotoxic effect, even after treatment with the maximal dosage of 20 µM.

### 3.5. Effect on Cell Cycle of Glioblastoma Cell Lines

**Figure 5:**
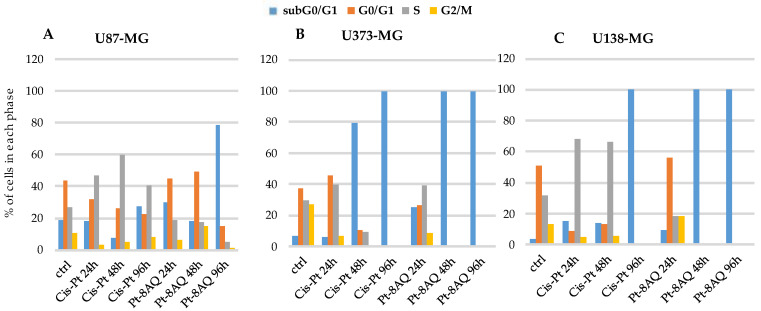
Effect of CisPt and Pt-8AQ on cell cycle on different glioblastoma cell lines after 24, 48, and 96 h. The histograms showed the effect of Pt-8AQ or CisPt treatments (10 µM) on U87-MG (**A**), U373-MG (**B**), and U138-MG (**C**) after 24 h, 48, or 96 h of treatment. The data were expressed as percentages of cells counted in each different cell phase (subG0/G1, G0/G1, S, and G2/M) compared to those found in untreated cells (CTRL). The values are expressed as mean of two replicates.

The effect of CisPt and Pt-8AQ on cell cycle in the three glioblastomas cells was evaluated at 24, 48, and 96 h after treatment at a drug concentration of 10 µM (Figure 5). All the glioblastomas treated with CisPt showed a significant decrease of the cells in G0/G1 phase (U87-MG: from 43% to 26%; U373-MG: from 37 to 10%; and U138-MG: from 50 to 13%) and a dramatic increase of cells in S phase (U87-MG: from 27% to 60%; U373-MG: from 30% to 40%; and U138-MG: from 31% to 66%) after 24 or 48 h of treatment.

At 96 h, the 100% of U138-MG cells and U373-MG were detected as an apoptotic population in subG0/G1 region. Pt-8AQ evidenced a higher apoptotic activity expressed by 80% of U87-GM cells in subG0/G1 after 96 h and 100% of U373-GM and U138-GM in subG0/G1 after only 48 h. These data are consistent with the known mechanism of inhibition of cell proliferation exerted by CisPt that is mediated by an S-phase arrest. Conversely, Pt-8AQ seemed to affect the proliferation by a prevalent early cell death mechanism able to overcome the block of cell cycle checkpoints and that it could be predictive for some different mechanisms by which this new compound affected glioblastoma cell growth.

### 3.6. Pro-Apoptotic Gene Expression and Apoptosis in Glioblastoma Cell Lines

**Figure 6:**
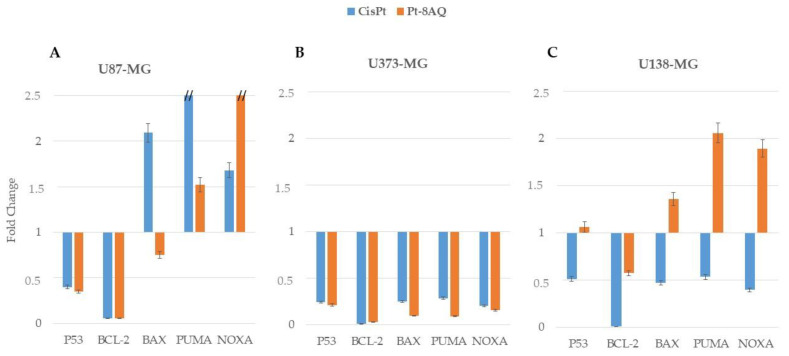
Rt-qPCR. RTq-PCR of proapoptotic molecules induced by p53 (PUMA, NOXA, and BAX) and antiapoptotic marker BCL-2 on glioblastoma cell lines U87-MG (**A**), U373-MG (**B**), and U138-MG (**C**) after 24 h treatment with CisPt or Pt-8AQ at 10 µM. The values are expressed as mean ± standard deviation (SD) of two replicates.

To determine the relation between the observed cytotoxic effects and the apoptosis-mediated cell death, we investigated the expression of p53 in glioblastoma cells treated with CisPt or Pt-8AQ drugs. As reported in Figure 6 at 24 h of treatment, both the drugs produced a significant downregulation of p53, with the only exception of U138-MG cells upon treatment with Pt-8AQ.

Other proapoptotic molecules transcriptionally induced by p53, such as *PUMA*, *NOXA*, and *BAX*, belonging to the Bcl-2 family, were considered during our investigation concerning the apoptosis pathway (Figure 6). RT-qPCR revealed that BCL-2, an antiapoptotic gene, was significantly downregulated both by CisPt and Pt-8AQ in all the treated glioblastoma cells. By considering U87-MG cells, CisPt treatment strongly upregulated *PUMA* and *BAX*, and to a minor degree, *NOXA*, whereas Pt-8AQ treatment induced a strongly expression of *NOXA*, a minor expression of *PUMA*, and *BAX* resulted in being downregulated. In U138-MG cells, the three genes were downregulated by CisPt and upregulated by Pt-8AQ. In U373-MG, all the three genes were significantly downregulated both by CisPt and Pt-8AQ.

**Figure 7:**
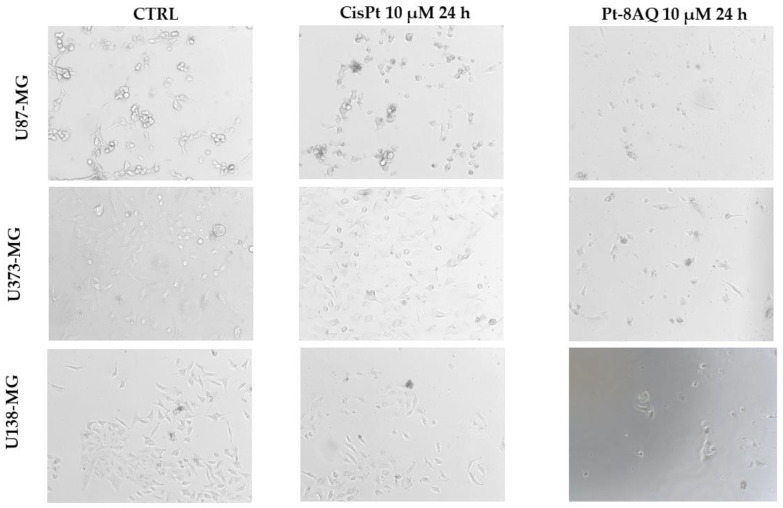
Morphological images of glioblastoma cell lines treated with CisPt and Pt-8AQ. Representative cells images at light microscope: control cells (CTRL) and cells treated 24 h with CisPt or Pt-8AQ 10µM (magnification 100×).

To better highlight the different cytotoxic effects of CisPt and Pt-8AQ on glioblastoma cell lines U87-MG, U373-MG, and U138-MG, we performed representative photographs at light microscope of untreated (CTRL) or treated cells for 24 h with CisPt or Pt-8AQ at 10 µM. As shown in Figure 7, cells treated with CisPt did not show consistent changes compared to the control cells, with only a few groups of cells detached showing some sign of damages. Cells treated with Pt-8AQ after 24 h were extensively detached from the surface and showed a completely altered morphology typical of significant cell damages. Many cellular debris were visible above all in U373 and U138 samples, underling the high cytotoxic effect exerted by Pt-8AQ.

**Figure 8:**
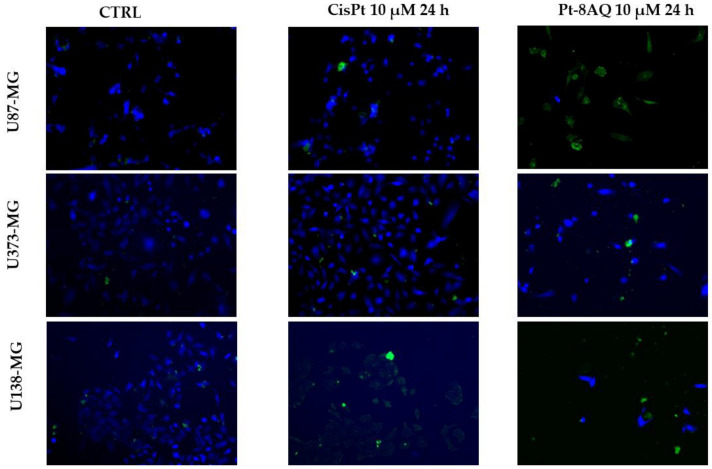
Viability assay on glioblastoma cell lines treated with CisPt and Pt-8AQ. Representative fluorescence images of control cells (CTRL) and cells treated 24 h with CisPt or Pt-8AQ 10µM. Healthy viable cells were stained with CytoCalcein Violet 450 (blue), and apoptotic cells with phosphatidylserine (green). No necrotic cells are present (7-aminoactinomycin D, red). (Magnification 100×).

To ascertain cell death mechanism and the degree of cellular damage, we performed triple staining with phosphatidylserine, 7-aminoactinomycin D, and CytoCalcein Violet 450 in U87-MG, U373-MG, and U138-MG treated 24 h with CisPt or Pt-8AQ at 10 µM. This staining approach allows for simultaneous detection of apoptotic, necrotic, or healthy cells, and confirms what has been detected by using light microscope analysis. As shown in Figure 8, control cells were mainly viable (blue) and mostly adherent, whereas those treated with CisPt were early apoptotic (simultaneously green and blue). Pt-8AQ 10 µM treated cells were mostly detached and late apoptotic, showing green fluorescence. Only few adherent cells are still viable. No red fluorescence (necrotic cells) was visible.

The above observations related to apoptotic mechanisms were confirmed by measuring the caspase-3 activity through a colorimetric assay at 4 h after the drug treatment, proving an increased activity of about 1.3 and 2.2 for the CisPt and Pt8AQ, respectively (data not reported).

## 4. Discussion

Our results evidenced that both CisPt and Pt-8AQ exerted a significant in vitro anticancer activity against all the evaluated cancer cells lines (Figure 1), although CisPt showed a higher activity than Pt-8AQ on both the tested carcinoma and mesothelioma. The most interesting observation concerns the glioblastoma cell models (U87-MG, U373-MG, and U138-MG), on which Pt-8AQ was more active than CisPt with a significant (*p* < 0.05) difference in terms of IC_50_ value. It is important to remark that U87-MG is p53 wild type and the other two GBM cell lines are p53 mutants. Glioblastoma is a very frequent and malignant brain tumor developed in adults with an etiology not yet clarified for which, according to our knowledge, the main treatment is based on radiotherapy associated with temozolomide, although unfortunately associated with poor results [23]. As recently reported, CisPt has been suggested as a possible adjuvant therapy in gliomas [24], and, in this perspective, our data on Pt-8AQ were found to be extremely significant and stimulated further investigations to define the pharmacological profile of this new compound. Indeed, preliminary studies to better clarify the stability and the activity of Pt-8AQ in comparison to CisPt were performed. As is known, platinum-based drugs have a limited solubility in culture medium, and they can also suffer from degradation process by pH and temperature modification [23], affecting the use of these compounds in chemotherapy. We found that, after 24 h treatment at 37 °C, Pt-8AQ stability was significantly higher than that of CisPt, considering that within this time, CisPt completely lost its activity when tested against U87-MG tumor cells line. Pt-8AQ, on the contrary, maintained a remarkable anticancer activity up to 6 days of treatment at 37 °C (Figure 2). The ability of the two drugs to affect the glioblastoma cell lines proliferation was further investigated by studying the effect on cell cycle after 24, 48, and 96 h of treatment. The results showed the ability of CisPt to arrest the cell cycle in S phase in agreement with the yet described mechanism of CisPt inducing DNA damage by starting from a transient S-phase arrest, followed by inhibition of Cdc2-cyclin A or B kinases to yield to a persistent G2/M arrest [25].

Only a partial low modulation of S phase cell cycle was significantly induced by Pt-8AQ in all the glioblastomas that, however, showed off a dramatic high percentage of cells in the SubG0/G1, suggesting that this molecule could affect proliferation and viability with a different mechanism as already previously observed for other cancer cell lines [8], and that Pt-8AQ could affect the proliferation by a prevalent early cell killing mechanism that overcomes the block of cell cycle checkpoints. In fact, if cells enter apoptosis from the S or G2/M phase of the cell cycle, they may not appear in the SubG1 peak. This idea is in agreement with the results of the direct cytotoxicity data that confirmed a different degree of action exerted by CisPt and Pt8AQ on the glioblastoma cells viability after 24 h of treatment with increasing dosages (Figure 4). As appreciable in all GBM lines, Pt-8AQ produced an early cell death with a higher cell mortality (24 h at 20 µM), whereas at the same dosage, CisPt did not affect cell viability. Moreover, our investigation on apoptosis (Figure 6 and Figure 7) showed that the 24 h treatment with CisPt at 10 µM (Figure 7) produced in all the GBMs only few cells with signs of damage (Figure 6), with few apoptotic signals detectable by fluorescence microscopy (Figure 7). On the contrary, a higher number of apoptotic cells (with rare cells in necrosis) were observed after Pt-8AQ treatment. This early apoptosis pathway in cells treated with Pt-8AQ was also confirmed by the higher increase in caspase-3 activity, as measured by colorimetric assay.

All the above data considered together seemed to suggest that CisPt’s effect on GBM cell proliferation devoid of early cell death could be explained by hypothesizing limited damages on DNA. Indeed, as reported by some authors [26], the initial arrest in S phase of the cell cycle induced by CisPt could exert a cytoprotective effect both by allowing repair mechanisms to re-establish DNA integrity and also by preventing potentially abortive or abnormal mitoses. If our hypothesis is correct, we could assume that in cells treated with CisPt, the effect on cell proliferation is followed by a delayed induction of cell death. On the contrary, the antiproliferative effect of Pt-8AQ, rather than correlated with the modulation of cell cycle, should be the result of the strong cytotoxic effect, probably consequential of an early apoptotic mechanism. Undoubtedly, other cellular death pathways cannot be excluded, because in the sub G0/G1 region, cells can be detected whose cause of death can be ascribed to different mechanisms (e.g., autophagy, oncosis, mitotic catastrophe, aptonecrosis), and further investigations could be of help to understand more about the mechanism due to Pt-8AQ.

Since it is well known [27] that p53 is one of the most important genes in human cancer, having a critical function during the cancer steps that drive neoplastic transformation and playing an important role in triggering apoptosis, we also examined p53 expression in the selected glioblastoma cell lines (one p53 wild-type and two p53 mutated) after treatment with the CisPt and Pt-8AQ. We found that both Pt-8AQ or CisPt at 24 h induced a significant downregulation of both p53 and anti-apoptotic gene Bcl-2 (Figure 6). The only exception was the poor or absent p53 modulation by Pt-8AQ in U138-MG, probably as a result of the specific p53 mutant state of this line. In any case, taken together, these data showed a good homogeneity of response in the three glioblastomas, especially concerning the dramatic down modulation of Bcl-2 that is consistent with the apoptotic mechanisms producing the observed cellular damages. In response to an acute cellular stress (e.g., a DNA damage), a downregulation of anti-apoptotic Bcl-2 gene was observed, having an important role in promoting apoptotic cell death by an upregulation of genes such as *PUMA*, *NOXA*, and *BAX*.

As we have found in the selected three cell models, the regulation of these genes is often contradictory, depending on the type of cell line. CisPt treatment clearly upregulated *PUMA* and *BAX*, as well as, to a minor degree, *NOXA*, in U87-GM, producing instead a downregulation of these genes in U138-GM in which, on the contrary, Pt-8AQ treatment induced a strongly expression of *NOXA* with a minor expression of *PUMA* and *BAX*. In fact, as reported by Yakovlev [28], in some cell models, the *NOXA* gene upregulation by p53 could trigger apoptosis without the role of *BAX*. Thus, the apoptotic pathway following CisPt treatment could be probably related to the involvement of *PUMA* and *BAX*, although, as previously reported [29], *PUMA* and *NOXA* can participate in a different dual pathway to the induction of apoptosis being *PUMA* (but not *NOXA*) activity switched on by calcium release from the endoplasmic reticulum and the subsequent caspase activation through a collateral specific pathway. Surprisingly, in U373-MG, all the three genes were significantly downregulated both by CisPt and Pt-8AQ. In our models, while the role of apoptotic genes such as *PUMA* and *NOXA* seemed to be important both in wild-type p53 cells and in the p53-mutated U138-MG cell line, the dramatic significant downregulation of all genes in U373-MG cell line still has to be understood. Of course, some of the above observed discrepancy in gene expression can be related to the different p53 genetic status in the selected cell lines, and our present data are not sufficient to precisely establish the role displayed by these mutations, suggesting the need of more investigations.

Our observations, however, clearly highlighted the different mechanisms of action of the two drugs, as already proved by the spectroscopic study related to the interaction between Pt-8AQ with 9-EtG, Mets-7, and GSH (Figure 3A). In fact, these data confirmed that DNA is not the main target of the hindered cationic complex Pt-8AQ, because only after 30 h did a significant peak appear at 8.64 ppm in ^1^H-NMR spectrum, if compared to the kinetics of CisPt-DNA interaction [30]. Furthermore, the study of sequestration by Mets-7 confirmed a long kinetics required for Pt-8AQ to interact with this methionine-rich motif octapeptide, underlining the limited sequestration process affecting Pt-8AQ and its ability to escape from this resistance mechanism. Finally, we found that the interaction between Pt-8AQ and GSH occurred in a shorter period of time (12 h) by confirming the Lewis basic behavior of -SH groups in the presence of the soft platinum metal center. As GSH is present in significant amounts in many types of different cancer cells, this molecule plays an important role in maintaining their oxidative balance, also offering a protective shield against xenobiotics. As reported by some authors, GSH could be also associated with the cisplatin resistance [31,32]. However, our investigation on the effect of CisPt and Pt-8AQ performed on U87-MG cells in the presence of high amounts of GSH (from 6.25 µM to 800 µM) did not evidence any reduction in their anticancer activity (data not reported). Indeed, it is also necessary to take into consideration that although GSH has been described by many authors as able to produce resistance to CisPt, this topic is still under debate, and according to some NMR studies [33], high GSH levels are not always correlated to a decrease in sensitivity to CisPt. At this stage of our study, we think that the most important conclusion is the clear dramatic efficacy of this new molecule (Pt-8AQ) exerted against glioblastomas independently of their p53 gene status and even its prominent stability at 37 °C. In a preliminary study (data not reported here), we confirmed the high resistance of mesenchymal stromal cells (MSCs) to Pt-8AQ, and, on the basis of our previous experience [34] gained on systems for cell*mediated drug delivery with CisPt, we are proceeding to obtain MSCs loaded with Pt-8AQ. This in principle could let us to realize a locally advanced cell therapy to overcome the systemic toxicity subsequent to CisPt treatment that is often a dose-limiting factor. A possible way to tentatively reduce its toxicity, as recently suggested [35], relies on an intra-tumoral administration of the drug, thus resulting effective within a narrow therapeutic window and paving the way for an efficient approach for glioma or other brain tumor treatments. We think that Pt-8AQ, locally delivered by MSCs at high concentrations within the tumor microenvironment and for a longer time if compared to other less stable antineoplastic agents, could be a more effective approach, as demonstrated in our previous studies [18,36].

## Figures and Tables

**Table 1:** Primers sequences for quantitative real-time polymerase chain reaction (RT-qPCR).

GENE	Sequence (5′-3′)	Ta (°C)
*GAPDH*	FW: GGAGTCAACGGATTTGGTCG REV: CTTCCCGTTCTCAGCCTTGA	60
*NOXA*	FW: GCTGGAAGTCGAGTGTGCTA REV: GGAGTCCCCTCATGCAAGTT	60
*BCL2*	FW: ATCGCCCTGTGGATGACTGAGT REV GCCAGGAGAAATCAAACAGAGGC	60
*PUMA*	FW: ATGCCTGCCTCACCTTCATC REV: TCAGCCAAAATCTCCCACCC	60
*BAX*	FW: TGGCAGCTGACATGTTTTCTGAC REV: TCACCCAACCACCCTGGTCTT	60
*P53*	FW: GAGCTGAATGAGGCCTTGGA REV: CTGAGTCAGGCCCTTCTGTCTT	60
